# The signs of computer tomography combined with artificial intelligence can indicate the correlation between status of consciousness and primary brainstem hemorrhage of patients

**DOI:** 10.3389/fneur.2023.1116382

**Published:** 2023-03-27

**Authors:** Guofang Liu, Juan Sun, Shiyi Zuo, Lei Zhang, Hanxu Cai, Xiaolong Zhang, Zhian Hu, Yong Liu, Zhongxiang Yao

**Affiliations:** ^1^Department of Radiology, Second Affiliated Hospital, Army Medical University (Third Military Medical University), Chongqing, China; ^2^Department of Pain and Rehabilitation, Second Affiliated Hospital, Army Medical University (Third Military Medical University), Chongqing, China; ^3^Department of Physiology, College of Basic Medical Sciences, Army Medical University (Third Military Medical University), Chongqing, China

**Keywords:** computer tomography sign, primary brainstem hemorrhage, status of consciousness, artificial intelligence, hemorrhage volume, hemorrhage involving the ventricular system

## Abstract

**Background:**

For patients of primary brainstem hemorrhage (PBH), it is crucial to find a method that can quickly and accurately predict the correlation between status of consciousness and PBH.

**Objective:**

To analyze the value of computer tomography (CT) signs in combination with artificial intelligence (AI) technique in predicting the correlation between status of consciousness and PBH.

**Methods:**

A total of 120 patients with PBH were enrolled from August 2011 to March 2021 according to the criteria. Patients were divided into three groups [consciousness, minimally conscious state (MCS) and coma] based on the status of consciousness. Then, first, Mann–Whitney U test and Spearman rank correlation test were used on the factors: gender, age, stages of intracerebral hemorrhage, CT signs with AI or radiology physicians, hemorrhage involving the midbrain or ventricular system. We collected hemorrhage volumes and mean CT values with AI. Second, those significant factors were screened out by the Mann–Whitney U test and those highly or moderately correlated by Spearman’s rank correlation test, and a further ordinal multinomial logistic regression analysis was performed to find independent predictors of the status of consciousness. At last, receiver operating characteristic (ROC) curves were drawn to calculate the hemorrhage volume for predictively assessing the status of consciousness.

**Results:**

Preliminary meaningful variables include hemorrhage involving the midbrain or ventricular system, hemorrhage volume, grade of hematoma shape and density, and CT value from Mann–Whitney U test and Spearman rank correlation test. It is further shown by ordinal multinomial logistic regression analysis that hemorrhage volume and hemorrhage involving the ventricular system are two major predictors of the status of consciousness. It showed from ROC that the hemorrhage volumes of <3.040 mL, 3.040 ~ 6.225 mL and >6.225 mL correspond to consciousness, MCS or coma, respectively. If the hemorrhage volume is the same, hemorrhage involving the ventricular system should be correlated with more severe disorders of consciousness (DOC).

**Conclusion:**

CT signs combined with AI can predict the correlation between status of consciousness and PBH. Hemorrhage volume and hemorrhage involving the ventricular system are two independent factors, with hemorrhage volume in particular reaching quantitative predictions.

## Highlights

- The signs of computer tomography (CT) combined with artificial intelligence (AI) can indicate the correlation between status of consciousness and primary brainstem hemorrhage (PBH) of patients.
- Hemorrhage volume and hemorrhage involving the ventricular system are two independent indicators of the status of consciousness in PBH patients.
- The hemorrhage volumes of <3.040 mL, 3.040 ~ 6.225 mL and >6.225 mL in PBH patients correspond to consciousness, minimally conscious state (MCS) or coma, respectively.

## 1. Introduction

The primary brainstem hemorrhage (PBH) accounts for 6–10% of intracerebral hemorrhages (ICH), with an incidence of approximately 2–4/100,000 per year, and approximately 60–80% of PBH occurring in the pontine region (1). The most severe and commonly clinical manifestation is disorders of consciousness (DOC) (2), and even death. Due to the acute onset and rapid progress of spontaneous PBH, we should comprehensively and carefully assess the characteristics of the patient’s status at an early stage to provide first-hand evidence for diagnosis and therapy.

To assess the status of consciousness should depend on the patient’s basic conditions, including the wake–sleep cycle, limb and eye movements, etc. Currently, the main evaluation methods mainly include (3): (1) Clinical examination, being divided into clinical symptom detection and behavior scales. Clinical symptom detection should identify confounding factors such as cortical blindness, endotracheal intubation, appropriate environment, and the acute or convalescent phase of the disease. Behavior scales should assess the status of consciousness mainly includes Glasgow coma scale (GCS) and Full outline of unresponsiveness (FOUR). (2) Electroencephalogram (EEG) tests, mainly including standard EEG and consciousness paradigm EEG. (3) Neuroimaging tests, which mainly include functional magnetic resonance imaging (fMRI) and positron emission tomography (PET). The fMRI and PET are mainly applied to patients who are unable to cooperate with clinical physical examinations and can effectively complete image collection with high quality and high standards. In addition, new techniques such as diffusion tensor imaging (DTI) (4) and diffusion tensor tractography (DTT) (5) are also being used to assess the status of consciousness.

All of the above methods for evaluating the status of consciousness are clinically applied and recommended, but the reliability of these evidence is only weak or moderate (3), and there are many disadvantages to them. They have different shortcomings in practical applications. For example, in some patients with severe supratentorial ICH, clinical indications, status of consciousness and quantitative EEG (qEEG) indicate that the patient is in a state of exhaustion (6). Continuous bedside EEG is only available in a select number of treating intensive care units (ICUs) (7), and the measures investigated, to date, are still experimental. Other forms of brain monitoring such as functional EEG (fEEG) and fMRI have been used to test for status of consciousness directly by using mental (motor or spatial) imagery tasks (8–11) or local–global paradigms (12, 13), but these require patient’s participation, it can only be performed intermittently, and is experimental and thus is not well suited to serve as alarm triggers. The limitations of PET scanners currently include long scanning time, low signal-to-noise ratio, and high dose of ionizing radiation (14). For PBH patients, consciousness assessment is an important event in the subsequent diagnosis and treatment, which can affect the choice of surgery (15) and judgment of patient’s prognosis (16), so it is crucial to find a method that can quickly and accurately indicate the correlation between status of consciousness and PBH.

The signs of computer tomography (CT) and structural magnetic resonance imaging (MRI) can reflect the sites, hemorrhage volume, and hemorrhage involving the ventricular system, and so on. Because the structural MRI tests take a long-lasting time (17), it is not suitable for PBH patients with altered consciousness, who may not be able to cooperate with the inspection for a long time. In contrast, CT scanning is routinely considered a preferred method for PBH assessment due to its general accessibility and rapid availability (2). In DOC patients, CT signs are now primarily used as an adjunct to diagnosis of disorders such as cerebral apoplexy, brain tumors, intracranial infections, and hepatic encephalopathy. CT scan has an advantage over diagnosing ICH, because they can show high density shadows in ICH patients. However, this is an observational and qualitative judgment based on CT signs in ICH patients, and quantitative diagnosis based on CT signs is now difficult. But with the progress of artificial intelligence (AI) technique, the traditional CT scan has developed new possibilities in diagnosing ICH (18). The application of AI technique in ICH mainly includes four aspects (19): etiological diagnosis, division of ICH, predicting the progression and prognosis of ICH. For example, the DEEPWISE Medical Artificial intelligence auxiliary diagnostic software has been proven reliably to measure hemorrhage volume which belongs to “division of ICH” (15).

In this study, we retrospectively collected and analyzed CT signs of PBH patients to quickly estimate the correlation between status of consciousness and PBH. We took four steps to complete this study. First, we collected a total of nine metrics that were analyzed by the radiologist in conjunction with the AI technique: gender, age, stages of intracerebral hemorrhage, hemorrhage volume, mean CT value, hemorrhage involving the midbrain, hemorrhage involving the ventricular system, grade of hematoma shape, and grade of hematoma density. Second, we performed a univariate analysis of the above factors to find the factors most likely to be related to the status of consciousness. Six factors were found to be significant. Then, to find factors that can indicate the status of consciousness, an ordered multivariate logistic analysis is performed on these six factors. Ultimately, we find two indicators (hemorrhage volume and hemorrhage involving the ventricular system) can indicate the status of consciousness in PBH patients. In addition, we drew the receiver operating characteristic (ROC) curve to quantitate hemorrhage volume and their relationship with the status of consciousness. The hemorrhage volumes of <3.040 mL, 3.040 ~ 6.225 mL and >6.225 mL in PBH patients were found to correspond to consciousness, minimally conscious state (MCS) or coma, respectively. It not only helps in the diagnosis and therapy of PBH, but also serves as a reference for multi-modal assessment of DOC.

## 2. Materials and methods

### 2.1. Inclusion criteria and exclusion criteria

We retrospectively collected and analyzed 390 patients with brainstem hemorrhage admitted to Department of emergency and Department of neurosurgery of our hospital from August 2011 to March 2021, and selected 120 PBH patients who met the inclusion and exclusion criteria as follows.

Inclusion criteria include that: (1) All patients were diagnosed with PBH (20) and underwent CT scan. (2) All patients were professionally evaluated for the status of consciousness by clinical manifestation. (3) All patients had not had surgery before the CT scan. (4) CT images were suitable for diagnosis, that is, all of the brainstem structures were included in the CT scanning range, and the CT images were clear without artifacts. (5) All patients must carry out both consciousness assessment and CT scanning, and the intervals between consciousness assessment and CT scanning were fixed [less than 0.5 h for emergency patients; less than 3 h for the hospitalized patients, it was based on the actual medical conditions of our hospital and literature reports to determine the time interval (21)].

Exclusion criteria (20) include: (1) Secondary brainstem hemorrhage. (2) PBH in association with underlying conditions that alter consciousness, such as severe diabetes, kidney failure, hepatic cirrhosis, cachexia, etc. (3) PBH in combination with altered consciousness due to medication, poisoning, etc. (4) A complex history of other brain-parenchymal disease in PBH patients. (5) PBH combined with hemorrhage in other parts of the brain parenchyma.

All procedures performed in this study involving human participants were in accordance with the Declaration of Helsinki (as revised in 2013). The study was approved by the Medical Ethics Committee of the Second Affiliated Hospital, Army Medical University (2021-Scientific research NO. 089-01). Individual consent from all patients was waived for this retrospective analysis.

### 2.2. Image acquisition

Craniofacial cross-sectional CT scans were performed in all patients with the orbitomeatal baselines as baselines and a scan range from the cranial apex to the cranial base. The sequence parameters are as follows: field of view = 2.5 cm; the layer thickness = 1.5 mm/5 mm/10 mm. CT models include: TOSGIBA-Aquilion one 640 (Dynamic Volume CT), GE-Optima CT 660 (128 T), GE-Lightspeed VCT-64. CT data complied with DICOM (digital imaging and communications in medicine) standard.

### 2.3. Clinical assessment and grouping of the status of consciousness

We rated the status of consciousness by Coma Recovery Scale-Revised (CRS-R) (30), and based on the literature (22). The PBH patients were divided into three groups according to the status of consciousness, that is, consciousness, MCS and coma. The “consciousness” group means that wakefulness and awareness are normal. The “MCS” group implies the inclusion of waking and fluctuating awareness with reproducible, purposeful behavioral responses to external stimuli. The “coma” group is one in which there is no wakefulness, no awareness of self or environment.

### 2.4. CT sign analysis and data acquisition

An associate chief radiologist and an attending radiologist independently analyzed the CT signs and were analyzed by Kappa (0.6 < Kappa < 0.85, *p* < 0.05), finally came to an agreement.

According to whether hemorrhages were involved in the ventricular system (23) or midbrain (24), the patients were further divided into two groups respectively: hemorrhage involving the ventricular system and hemorrhage not involving the ventricular system; hemorrhage involving the midbrain and hemorrhage not involving the midbrain.

Two major imaging characteristics are the variation of hematoma shape (irregular versus regular) and hematoma density (homogeneous versus heterogeneous). Conceptually (25), hemorrhage arising from a solitary focus should tend to present a more regular hematoma shape, and a more homogeneous hematoma density; and hemorrhages resulting from multiple foci should be more likely to present an irregular hematoma shapes and a more heterogeneous hematoma densities. Heterogeneous hemorrhages are potentially fed by multiple bleeding intracerebral vessels, resulting in a mixture of hematologic components, including fresh hemorrhages with low attenuation of fluid and stale hemorrhages with high attenuation of blood clots.

**Figure 1:**
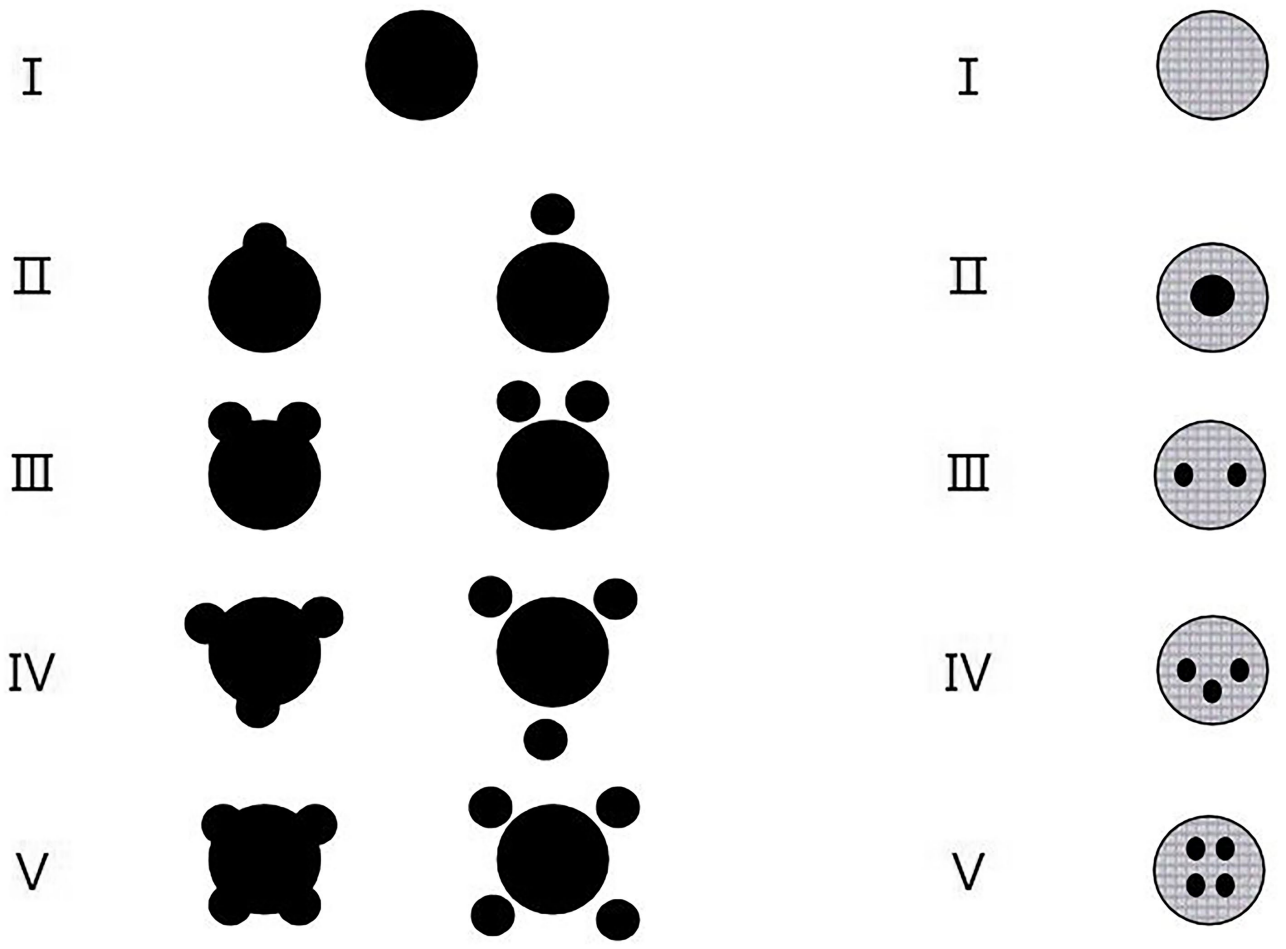
The categorical scales according to the grades of hematoma shapes (left lane) and the grades of hematoma densities (right lane). (Left lane) Classification of hematoma shapes by grade. Grade I, Only one regular (usually rounded) hematoma; Grade II, One regular hematoma plus one small hemorrhagic satellite focus; Grade III, One regular hematoma plus two small hemorrhagic satellite foci; Grade IV, One regular hematoma plus three small hemorrhagic satellite foci; Grade V, One regular hematoma plus four or more small hemorrhagic satellite foci. Regular hematoma shapes include grades of I and II while irregular hematoma shapes include grades of III to V. (Right lane) Classification of hematoma densities by grade. Grade I, Macroscopic hematoma density is consistent; Grade II, Macroscopic hematoma density are segmented to 2 places; Grade III, Macroscopic hematoma density are segmented to 3 places; Grade IV, Macroscopic hematoma density are segmented to 4 places; Grade V, Macroscopic hematoma density are segmented to 5 places or more. Homogeneous hematoma densities include grades of I and II while heterogeneous hematoma densities include grades of III–V.

We analyzed hematomas using a categorical scale based on classification of hematoma shape and hematoma density (25) (Figure 1). The grades of hematoma shapes are described as I-V and further divided into two groups, that is, regular (I–II) and irregular (III–V) groups (Figure 1, left lane). The grades of hematoma densities are also described as I–V and further divided into two groups, that is, homogeneous (I–II) and heterogeneous (III–V) groups (Figure 1, right lane).

In a hematoma, the hemoglobin mainly passes through three forms (oxyhemoglobin, deoxyhemoglobin, and methemoglobin) prior to red cell lysis and is break down into ferritin and hemosiderin. Based on these forms of hemoglobin, we can define four distinct stages of intracerebral hemorrhage: hyperacute, acute, subacute and chronic (26).

In addition, we analyzed the raw CT images in DICOM (digital imaging and communications in medicine) format for all cases of cerebral hemorrhage using DEEPWISE Medical Artificial intelligence auxiliary diagnostic software (15) (Type: Web-PANS Version: 1.0.1.1). We collected hemorrhage volumes (hemorrhage volume only in brainstem parenchyma was counted, and blood accumulation in other parts such as ventricular system was not counted) and CT values. The AI system calculated hemorrhage volumes (single voxel point × rendered area × layer thickness). It directly extracted the mean CT values by CT instrument.

### 2.5. Statistical analysis

All data were analyzed using SPSS (statistical product service solution) 24.0, and statistical significance was defined as *p* < 0.05. Firstly, to seek factors that may affect the status of consciousness in PBH patients, we performed a univariate analysis that included Mann–Whitney U test and Sperman rank correlation test. According to the categories of statistical independent variables, the factors of independent variables were divided into two categories: ① The nominal data including gender, hemorrhage involving the midbrain or ventricular system, which were used by Mann–whitney U test (/r/ ≤ 0.2: no connections; 0.2 < /r/ ≤ 0.4: weakly; 0.4 < /r/ ≤ 0.6: moderately; /r/ > 0.6: highly). ② The ordinal data including age, grades of hematoma shape, grades of hematoma density, CT values, hemorrhage volumes, which were used by Sperman rank correlation tests.

Secondly, to find factors that can indicate the status of consciousness, an ordered multivariate logistic analysis was performed on the statistically significant variables screened in the previous step. In order to satisfy the conditions of ordered multivariate logistic analysis, the continuous variables were grouped in order.

Finally, in order to find the relationships between hemorrhage volumes and the status of consciousness in more details, we drew the ROC curve.

## 3. Results

### 3.1. The general data

**Figure 2:**
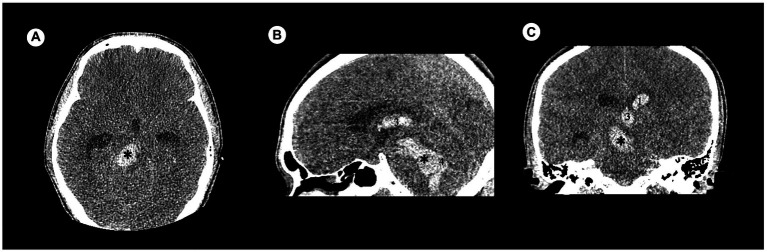
Examples of hemorrhage involving the ventricular system in patients with acute obstructive hydrocephalus detected by craniofacial computer tomography (CT) scans. **(A)** Transverse view: Hematoma was present in brainstem (✱). **(B)** Sagittal view: Hematomas were present in brainstem (✱), third ventricle (3), and fourth ventricle (4). **(C)** Coronary view: Hematomas were present in brainstem (✱), third ventricle (3), and lateral ventricle (1).

**Figure 3:**
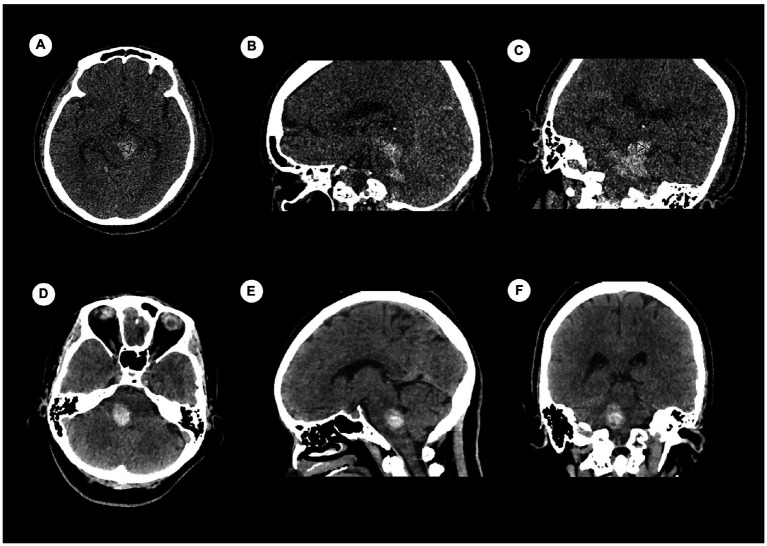
Examples of hemorrhage involving the midbrain (upper) and not involving the midbrain (lower) detected by craniofacial computer tomography (CT) scans. **(A)** Transverse view: Hematoma was present only in midbrain (▷). **(B)** Coronary view: Hematomas were present in midbrain (▷) and pons. **(C)** Sagittal view: Hematomas were present in midbrain (▷) and pons. **(D)** Transverse view: Hematoma was present only in pons. **(E)** Coronary view: Hematoma was present only in pons. **(F)** Sagittal view: Hematoma was present only in pons.

There were 120 cases with PBH, and the number of cases in different groups classified by the status of consciousness were 50 for consciousness, 26 for MCS and 44 for coma. There were 92 males and 28 females. Age ranged from 9 to 83 years, with a median of 52.85 years. Hemorrhages involving the ventricular system were 20 (Figure 2), and not involving the ventricular system were 100 (Figure 3); hemorrhages involving the midbrain were 73 (Figures 3A–C), and not involving the midbrain were 47 (Figures 3D–F). There were 39 patients in the hyperacute stage of intracerebral hemorrhage, 39 in the acute stage, 38 in the subacute stage and 4 in the chronic stage.

**Figure 4:**
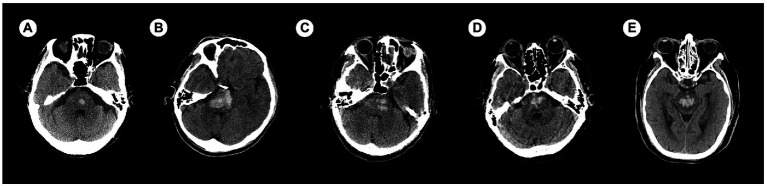
Examples of categories based on hematoma shape and density. **(A)** Category I-I (shape I—density I). **(B)** Category II-II (shape II—density II). **(C)** Category III–III (shape III—density III). **(D)** Category IV–IV (shape IV—density IV). **(E)** Category V–V (shape V—density V). The categories of I and II were defined as regular/homogeneous while the categories of III to V were defined as irregular/heterogeneous.

There were 44 in regular shapes (shape I 17, shape II 27), 76 in irregular shapes (shape III 19, shape IV 18, shape V 39); and 66 in homogeneous densities (density I 26, density II 40), 54 in heterogeneous densities (density III 28, density IV 14, density V 12). There were not always a one-to-one correspondence between the shape of a hematoma and its density. We selected some samples of CT images for demonstration (Figure 4).

**Figure 5:**
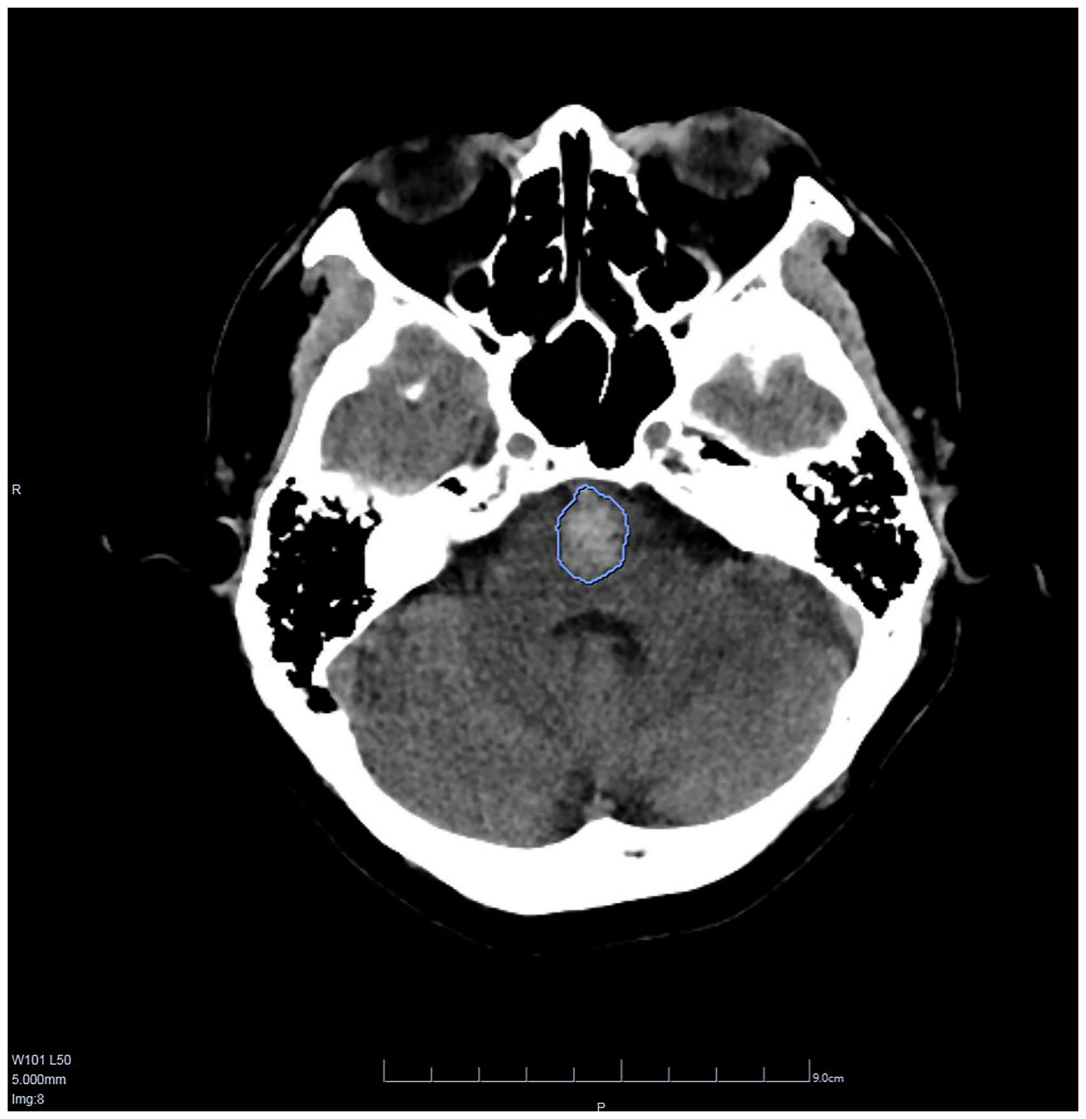
A screenshot of DEEPWISE Medical Artificial intelligence auxiliary diagnostic software. The hemorrhage volume in this patient was 3.97 mL and the CT value was 43.4 HU.

CT values and hemorrhage volumes were both analyzed by AI (Figure 5), and the median of hemorrhage volume was 6.4 mL (range: 0.06–39.37 mL). The median CT value was 45.5 HU (range: 27.7–55.9 HU).

### 3.2. Data analysis

#### 3.2.1. Initial screening for possible indicators of the status of consciousness

It showed that gender had no significant correlation with the status of consciousness (*p* > 0.05), while hemorrhage involving the midbrain or ventricular system were significant by Mann–Whitney U tests (*p* < 0.05; Table 1). In order to verify the correlations between the ordinal data and the status of consciousness, Spearman rank correlation tests were further adopted. The results showed that age was not correlated with the status of consciousness (*p* > 0.05, /r/ = 0.067), stages of intracerebral hemorrhage were weakly (*p* < 0.05, /r/ = 0.283), grades of hematoma shapes and densities and CT values were moderately (*p* < 0.05, /r/ = 0.415, /r/ = 0.430, /r/ = 0.419, respectively), and hemorrhage volume were highly (*p* < 0.05, /r/ = 0.742) correlated with the status of consciousness (Table 2).

**Table 1:** Correlation between nominal data and the status of consciousness by Mann–Whitney U test.

The nominal data	*P*	Significance
Gender	0.255	No
Hemorrhage involving the midbrain	0	Yes
Hemorrhage involving the ventricle system	0	Yes

**Table 2:** Correlation between ordinal data and the status of consciousness by Spearman rank correlation test.

The ordinal data	*P*	*r*	Connection levels
Age	0.468	0.067	No
Stages of intracerebral hemorrhage	0.002	−0.283	Weakly
Grades of hematoma shapes	0	0.415	Moderately
Grades of hematoma densities	0	0.43	Moderately
CT values	0	0.419	Moderately
Hemorrhage volume	0	0.742	Highly

/r/ ≤ 0.2: No connections; 0.2 < /r/ ≤ 0.4: weakly; 0.4 < /r/ ≤ 0.6: moderately; /r/ > 0.6: highly.

#### 3.2.2. Further identifying the main factors that indicate the status of consciousness

To further identify the factors that best indicate the status of consciousness, an ordered multi-class categorical logistic analysis was used. The significances include the Model Fitting Information (Link function: Logit. *χ*^2^ = 93.416, *p* < 0.05) and Test of Parallel Lines (*χ*^2^ = 6.811, *p* > 0.05).

It showed that: ① the main factors are hemorrhage volume [OR = exp. (β) = exp. (2.092) = 8.101101 > 1, *p* < 0.05] and hemorrhage involving the ventricular system [OR = exp. (β) = exp. (2.321) = 10.18586 > 1, *p* < 0.05]. ② there were no significant correlations among the status of consciousness and grades of hematoma shapes, grades of hematoma densities, CT values, hemorrhage involving the midbrain (Table 3).

**Table 3:** Order-based multi-class categorical logistic analysis on the CT signs of patients with primary brainstem hemorrhage (PBH) to indicate the status of consciousness.

							95% CI	
		Estimate	SE	Wald	df	Sig. (*P*)	Lower bound	Upper bound
The status of consciousness	Consciousness	5.571	1.059	27.67	1	0	3.495	7.647
	MCS	7.249	1.174	38.12	1	0	4.948	9.55
	Coma	0^a^	0^a^	0^a^	0	0^a^	0^a^	0^a^
CT signs	Hemorrhage involving the midbrain	0.44	0.478	0.847	1	0.357	−0.497	1.376
	Hemorrhage involving the ventricle system	2.321	0.944	6.047	1	0.014	0.471	4.17
	Grades of shapes	0.589	0.559	1.11	1	0.292	−0.507	1.686
	Grades of densities	0.69	0.505	1.864	1	0.172	−0.3	1.68
	CT values	0.496	0.437	1.29	1	0.256	−0.36	1.353
	Hemorrhage volume	2.092	0.501	17.43	1	0	1.11	3.074

SE, standard error; df, degree of freedom; Sig. (*P*), significance (*p-*values). ^a^This parameter is set to zero because it is redundant.

#### 3.2.3. Quantitative relationship between hemorrhage volume and the status of consciousness as measured by ROC curve

The basic thoughts are that the hemorrhage volumes were tested to conform if it passed the normal distribution test, then verify the pairwise differences between the hemorrhage volumes and the status of consciousness (that is, consciousness, MCS and coma), and the critical value of hemorrhage volume was further determined to lie between consciousness and MCS, and between MCS and coma.

##### 3.2.3.1. The hemorrhage volume following a naturally lognormal distribution

**Figure 6:**
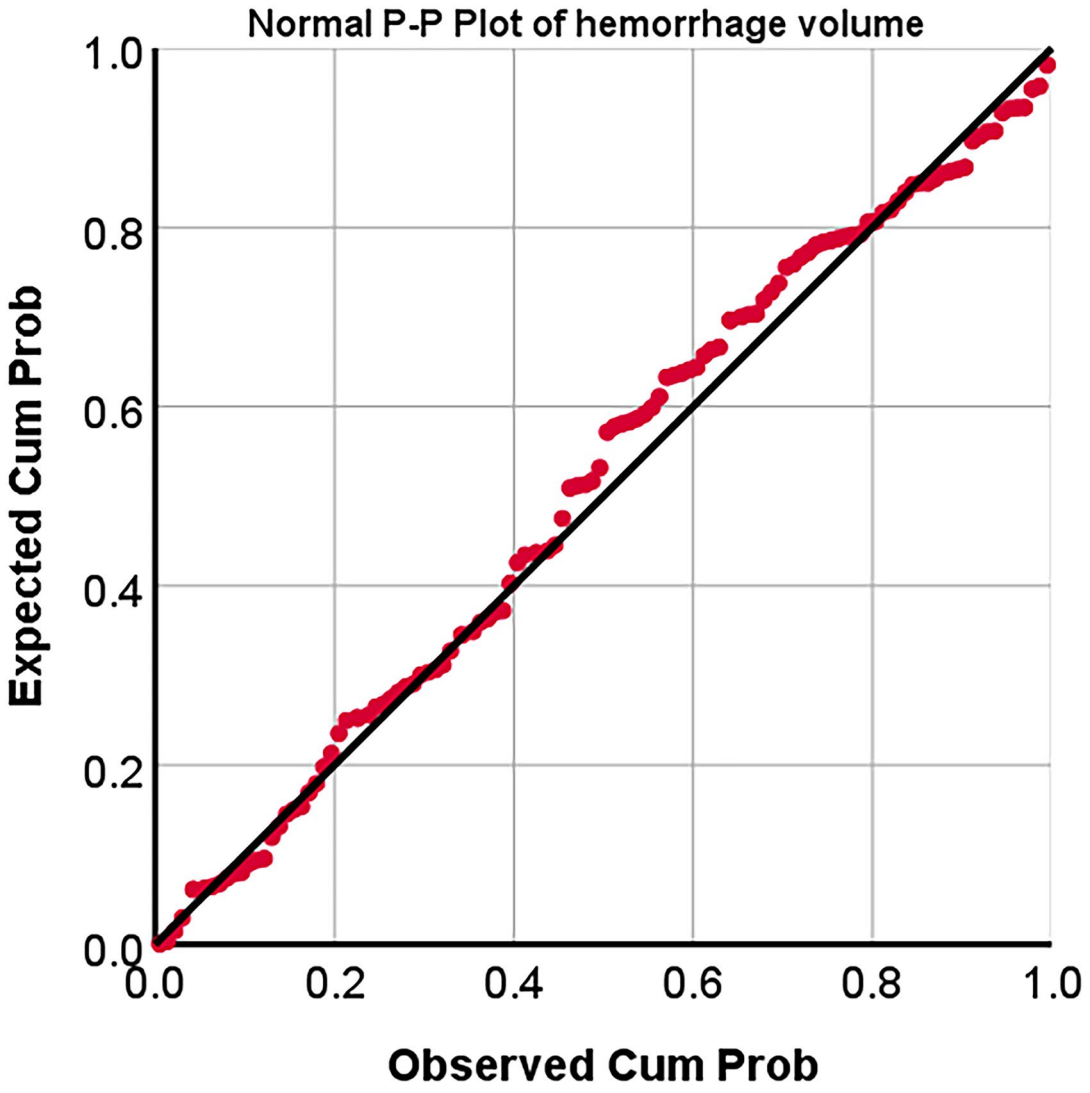
Normal probability-probability Plot (p–p plots) of hemorrhage volumes. P–P Plot, probability-probability Plot; Expected *cum* prob., Expected cumulation probability; Observed *cum* prob., Observed cumulation probability.

The probability-probability plots (P–P plots) were used to analyze the statistical characteristics of hemorrhage volumes, and it showed that the points in the P–P plots diagram are approximately a straight line. Thus, hemorrhage volumes accord with natural lognormal distribution (Figure 6).

##### 3.2.3.2. Pairwise differences in hemorrhage volume and the status of consciousness

One-way ANOVA (*F* = 56.332, *p* < 0.05) were conducted for the hemorrhage volumes and the status of consciousness, and Students-Newman–Keuls were used to compare them. It showed that there are pairwise differences (*α* = 0.05) among the hemorrhage volumes and the status of consciousness (that is, consciousness, MCS and coma) (Table 4).

**Table 4:** Pairwise differences in hemorrhage volume and the status of consciousness.

The status of consciousness	*N* (number of cases)	Subset for alpha = 0.05		
		1	2	3
Consciousness	50	0.50393765		
MCS	26		1.44712103	
Coma	44			2.26281379
Significance (*p*)		1	1	1

The Student–Newman-Keuls^a^^,^^b^ implication for groups in a homogeneous subset is shown. Type I error levels are not guaranteed.

a The harmonic mean sample size = 36.951 was used.

b The group sizes are not equal. The harmonic means of group size are used.

##### 3.2.3.3. The ROC curve plotting to show the indicative value of hemorrhage volume to the status of consciousness

**Figure 7:**
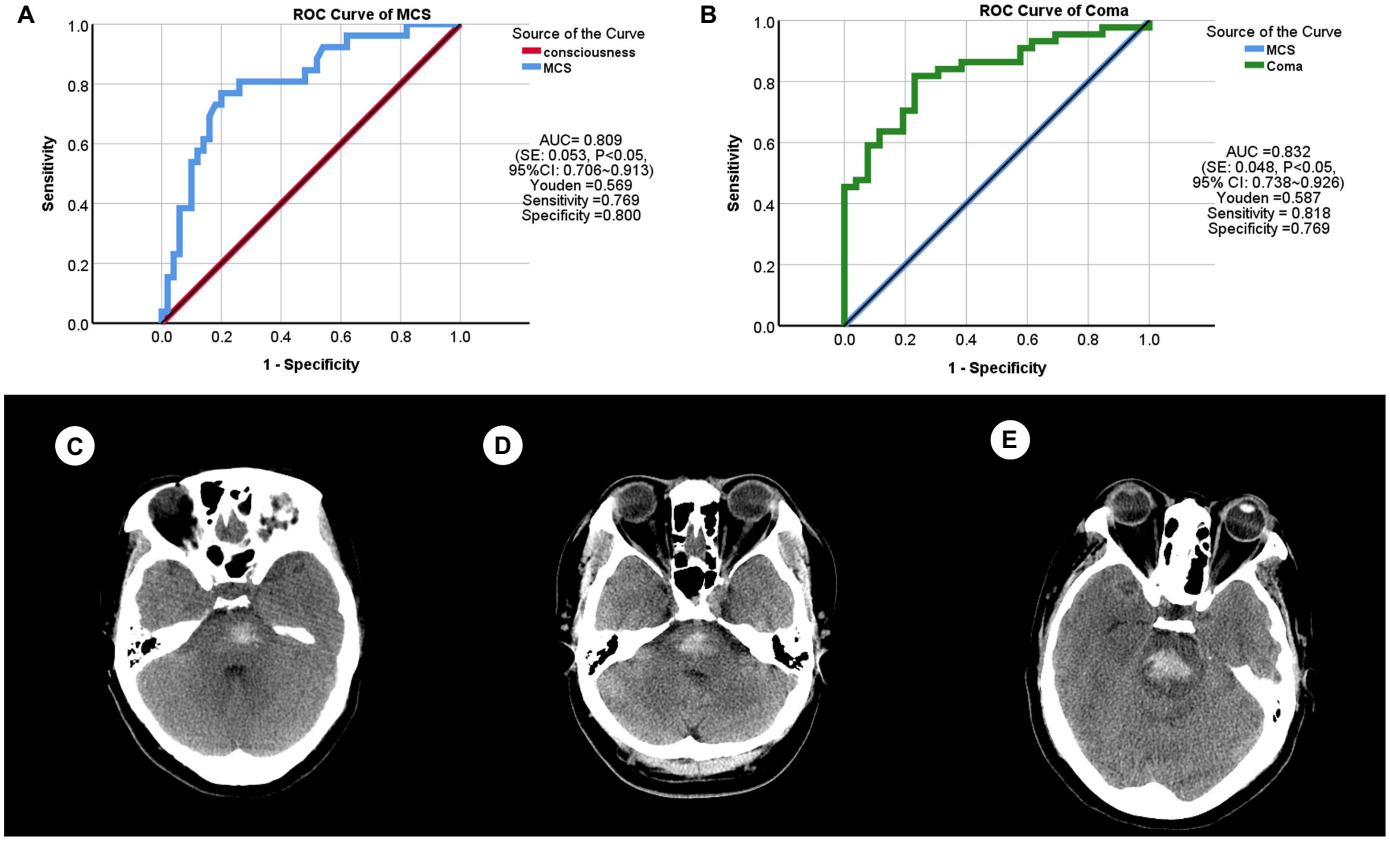
The receiver operating characteristics (ROC) curves and patient samples of primary brainstem hemorrhage (PBH). **(A)** ROC curve between consciousness and minimally conscious state (MCS). AUC (area under curve) = 0.809 [standard error (SE): 0.053, *p* < 0.05, 95% confidence interval (CI): 0.706 ~ 0.913], Youden = 0.569, sensitivity = 0.769, specificity = 0.800. **(B)** ROC curve between MCS and coma. AUC = 0.832 (SE: 0.048, *p* < 0.05, 95% CI: 0.738 ~ 0.926), Youden = 0.587, sensitivity = 0.818, specificity = 0.769. **(C)** This PBH patient (male, 40 years) was indicated to be consciousness and had a hemorrhage volume of 2.090 mL. **(D)** This PBH patient (female, 47 years) was indicated to be MCS with a hemorrhage volume of 4.820 mL. **(E)** This PBH patient (female, 51 years) was indicated to be coma with a hemorrhage volume of 6.950 mL.

In order to find the critically indicative value of hemorrhage volume between consciousness and MCS, we drew the ROC curve (Figure 7A) and it showed that the area under curves (AUC) is 0.809 (standard error (SE): 0.053, *p* < 0.05, 95% confidence interval (CI): 0.706 ~ 0.913), the Youden is 0.569, the sensitivity is 0.769, the specificity is 0.800, and the critical value is 3.040 mL.

In order to find the critically indicative value of hemorrhage volume between MCS and coma, we also drew the ROC curve (Figure 7B) and it showed that the AUC is 0.832 (SE: 0.048, *p* < 0.05, 95% CI: 0.738 ~ 0.926), the Youden is 0.587, the sensitivity is 0.818, the specificity is 0.769, and the critical value is 6.225 mL.

In short, PBH patients were indicated to be in consciousness when hemorrhage volume is less than 3.040 mL, to be in MCS when it is between 3.040 and 6.225 mL, and to be in coma when it is more than 6.225 mL.

To demonstrate these results, we randomly selected three patients with PBH (Figures 7C–E). Patient one (Figure 7C) came to Department of emergency with an eight-hour dizziness, and was in conscious and the hemorrhage volume was 2.09 mL. Patient two (Figure 7D) experienced sudden confusion and right limb weakness for 1 day, and was in MCS and the hemorrhage volume was 4.82 mL. Patient three (Figure 7E) could not be awakened for 12 h, and was in coma and the hemorrhage volume was 6.95 mL.

## 4. Discussion

Brainstem hemorrhage is a common serious disease and a common cause of acute or chronic DOC (2), with poor prognosis. In recent years, advances in CT signs combined with AI have made the diagnosis of ICH more accurate (18). Here, the CT signs of PBH patients are analyzed retrospectively by combining radiologists and AI in the hope of finding simple CT signs that indicate the correlation between conscious state and PBH and provide references for clinical diagnosis, therapy and scientific research.

In addition to the CT signs, we selected three other important fundamental clinical data, namely gender, age, and stage of intracerebral hemorrhage, for analysis. Because gender and age are risk factors for ICH (27). The determination of the stage of intracerebral hemorrhage is an important part of the application of CT in the diagnosis of hemorrhage from the temporal axis. But there was only a weak correlation between the stage of intracerebral hemorrhage and the state of consciousness. The stages of intracerebral hemorrhage are mainly based on the pathological basis and CT or MRI signs. Although CT signs are the first choice for the diagnosis of intracerebral hemorrhage, the stages of intracerebral hemorrhage were not mentioned in recommending imaging evidence in clinical treatment (28). So, its significance for clinical treatment needs to be further investigated. Here, we focus on the analysis of the relationship between CT signs and the state of consciousness.

The aim of this study is to find the most valuable CT signs for DOC diagnosis by analyzing routine CT signs of PBH patients. Radiologists generally analyze the signs of ICH, including the location of the hemorrhage, differences in shape, heterogeneity in density, CT values, hemorrhage volume, and whether the hemorrhage involves the ventricular system.

Based on CT signs, we found two of the most important indicators of the status of consciousness in PBH patients. Hemorrhage volume and hemorrhage involving the ventricular system are independent indicators of the status of consciousness. The hemorrhage volume was calculated using AI to avoid human errors (29), and hemorrhage involving the ventricular system is an objective and easily diagnosed by CT signs of ICH patients. These two CT signs are more direct and objective than the DOC assessment based on patient behavior in ICH patients.

Most previous studies have focused on the relationship between CT signs and prognosis for brainstem hemorrhage, and found that hemorrhage volume is an independent risk factor. Motivated by this, here we observed a quantitative relationship between hemorrhage volume and the status of consciousness in PBH patients. We can simply speculate the status of consciousness from the hemorrhage volume of PBH patients directly. When the hemorrhage volume is less than 3.040 mL, the patients should be conscious. Patient should be in MCS when the hemorrhage volume is between 3.040 and 6.225 mL, and when the hemorrhage volume is more than 6.225 mL, the patients should be in coma. Early treatment of PBH is mainly conservative treatment and surgical treatment (2). Identifying the optimal candidate for surgery is an essential problem. Through analyses of prognostic factors, Tao et al. (40) concluded that patients with a smaller hematoma (>5 mL and <10 mL), a greater GCS score (>6 and <8), age <65 years, unilateral tegmental hemorrhage, and without extrapontine extension might benefit from surgical treatment (40). Furthermore, based on their experience with five severe cases of surgical treatment, Shrestha et al. (41) proposed their indication for surgery: (1) hemorrhage volume >5 mL (concentrated relatively); (2) GCS score <8 with progressive neural dysfunction; (3) unstable basic vital signs, especially for patients who require mechanical ventilation; (4) location of the hematoma to <1 cm from the brainstem surface; and (5) time of hemorrhage with <24 h. There have also been scholars who have given hemorrhage severity scores based solely on the amount of hematoma and the state of consciousness (42), that is, <5 mL of hematoma volume was score 0, 5–10 mL was score 1, >10 mL was score 2; GCS 8–15 for 0, GCS 5–7 for 1 and GCS 3–4 for 2. Patients with total scores of 0, 1, 2, 3, and 4 had 30-day death rates of 2.7, 31.6, 42.7, 81.8 and 100%, respectively. The mortality rate of conservative treatment is extremely high for patients with massive brainstem hemorrhage or the brainstem hemorrhage severity score is >2. These patients are the primary target population for surgical procedures. In summary, the amount of bleeding and the state of consciousness were the two main factors in whether the operation was performed. In this manuscript, we analyze the correlation between the amount of bleeding and the state of consciousness. Therefore, we speculated that when it is “hemorrhage volume of 0–3.040 mL indicates consciousness,” conservative treatment can be selected [mainly including the use of anticoagulant drugs, etc. (28)]; when it is “above 6.225 mL means coma,” surgery may be a better choice; when it is “3.040–6.225 mL suggests MCS,” the treatment method was selected according to the comprehensive evaluation of bleeding site and vital signs.

Nowadays, CT techniques are widely used, but AI techniques are not widely used in primary hospitals. In practical clinical work, the ABC/2 formula is widely used in calculating the hemorrhage volume in cerebral parenchyma (31). Here, the AI software is also verified on the ABC/2 formula to calculate the hemorrhage volume (29). In addition, the hemorrhage volume calculated by AI is based on a CT thickness of 1.5 mm/5 mm/10 mm, which does not require a high configuration of CT instruments. Therefore, if high-end medical CT equipment or AI software is not available in primary hospitals, radiologists and clinicians can also use the ABC/2 formula to calculate the hemorrhage volume and give an accurate diagnosis with reference to this conclusion.

The brainstem is dorsally adjacent to the fourth ventricle and ventrally adjacent to the prepontine cistern, so hemorrhage from the brainstem can easily involve the ventricular system. When hemorrhage involves the ventricular system, a wide range of pathophysiological changes occur, most commonly acute obstructive hydrocephalus. Due to the sudden blockage of cerebrospinal fluid circulation, the brain ventricular system acutely enlarges in a few hours, and it should result in short-term or lasting vision changes, even can be prone to coma (32). In addition, it reported that if hemorrhage is involved in the ventricular system, the blood cells itself should lead to an inflammatory response and then impinge on the hypothalamus, and aggravate the status of consciousness (33). Here, we find that hemorrhage involving the ventricular system is a risk factor for worsening the status of consciousness in PBH patients. Therefore, in practical clinical work, no matter how large the hemorrhage volume is, when we need to judge the status of consciousness, we should refer to the CT signs to assess whether the hemorrhage is involved in the ventricular system.

Other factors, consisting of hemorrhage location, shape and heterogeneous density grades, as well as CT values, were not associated with indicating the status of consciousness. The midbrain and caudal diencephalon of brainstem are the key parts of the ascending reticular activating system (ARAS) (24). However, we find here that whether the midbrain is involved in hemorrhage is not correlated with the status of consciousness. The study showed that CT value could distinguish cerebral hemorrhage. The hematomas with average CT value <64 HU were mostly fresh bleeding, >80 HU was mostly coagulated clots (34). Clinically, the CT values from ICH can be used as a reference for the selection of surgical methods (34). Here, all the CT values of PBH patients are below 60 HU, which belongs to fresh blood, so it is pointless in judging the status of consciousness in PBH patients. We analyzed the static CT values and found that the CT values at these specific epochs are independent of the state of consciousness. However, the dynamic changes of CT values, although being associated with the status of consciousness, were still not to affirm its roles. It has been reported that it is significant decreased of the CT values of cerebral hemorrhage on the 5th day (35). In 80 of the 120 cases collected in our study, the bleeding duration was less than 5 days. Therefore, whether the dynamic CT value is related to the state of consciousness needs to be investigated further and may require collecting more cases. Differences in shape and heterogeneous density are the most common indicators of CT signs in PBH patients; however, they were not helpful in judging the status of consciousness in PBH patients.

Patients with hemorrhage in brainstem progress rapidly. Once diagnosed, it is necessary to quickly judge and assess the status of disease and immediately to undergo conservative or surgical treatment. The etiologies of DOC are diverse (36), such as poisoning (alcohol, carbon monoxide, etc.), cerebrovascular diseases (ICH, cerebral infarction, etc.), metabolic encephalopathy (respiratory failure, hypoglycemia, diabetic ketoacidosis, hepatic encephalopathy, etc.), cardiac arrest, intracranial infection and so on. Therefore, a differential diagnosis of the status of consciousness is important. If PBH is co-morbid with these diseases, we provide here an excellent reference for a comprehensive analysis of the patient’s condition.

In recent years, with rapid advances of AI techniques, image omics and machine learning, various prediction models have emerged with ICH patients (18, 37). Here, the CT signs of PBH patients were initially analyzed to provide a reference for an accurate indicative model, that should include hemorrhage volume and whether hemorrhage involving the ventricular system.

Previously, the CT sign was never included in the indication of the correlation between the state of consciousness and the PBH. Here, brainstem hemorrhage is selected to explore and evaluate the status of consciousness as a new perspective, namely CT signs. Currently, CT devices are widely used, which makes it easy to collect data and generalize. When we quantitatively evaluate the status of consciousness, it may be possible to find a new evaluation method based on etiologies and objective examination metrics.

In summary, hemorrhage volume and hemorrhage involving the ventricular system are independent factors in indicating the correlation between the state of consciousness and PBH. If the hemorrhage did not involve in the ventricular system, the status of consciousness can be preliminarily indicated according to hemorrhage volume, that is, hemorrhage volume of 0 ~ 3.040 mL indicates consciousness, 3.040 ~ 6.225 mL suggests MCS, and above 6.225 mL means coma. If hemorrhage volumes in PBH were the same, patients with involved in the ventricular system should become worse. If hemorrhage is involved in the ventricular system, the state of consciousness should become worse based on the magnitude of the hemorrhage as judged by the volume of hemorrhage. This provides a valid reference for the clinical diagnosis and therapies of PBH patients. Although, most of the major studies on ICH have focused on the relationship between the amount of ICH and prognosis, less attention has been paid to the correlation between conscious state and PBH. It has been reported that early coma is the prognostic factor of primary brainstem hemorrhage (38), indicating that it is of clinical significance to judge the status of consciousness as soon as possible. Given the ever-evolving precision medicine framework that integrates valuable behavioral assessment tools, sophisticated neuroimaging and electrophysiological techniques, a considerably higher diagnostic accuracy rate for DOC may now be attainable. Clarifying the mechanisms of consciousness, constructing unique and clinically precise identification techniques, and improving arousal rates remain difficult tasks to be solved. So, we should never neglect any type of methods which should evaluate more verifiable (39). Here, this sets a precedent for assessing the status of consciousness with CT signs and provides new ideas for future studies of DOC.

## 5. Limitations and further directions

The correlation between conscious state and PBH is only one-sided when only CT landmarks are used to represent it. There are many causes associated with impaired consciousness in PBH patients, such as basic vital signs, electrolytes, etc. The incidence of PBH is relatively low and it is difficult to collect surgical data from a large sample over a short period of time. Patients with PBH are in critical condition and some die immediately due to rapid brainstem failure. Rapid assessment of awareness is an important event in diagnosis and treatment, and CT scanning is the method of choice for PBH diagnosis. The analysis of the state of consciousness through the analysis of CT landmarks is certainly the most direct and simple approach. In the future, more methods, such as EEG and biochemical indicators, will be selected to comprehensively assess the correlation between the state of consciousness and PBH and thus aid in clinical diagnosis and treatment.

## Data availability statement

The raw data supporting the conclusions of this article will be made available by the authors, without undue reservation.

## Ethics statement

The studies involving human participants were reviewed and approved by the Second Affiliated Hospital, Army Medical University (Third Military Medical University), Chongqing 400037, China. Individual consent from all patients was waived for this retrospective analysis.

## Author contributions

GL collected, extracted, analyzed, interpreted the materials, and wrote the manuscript. JS, SZ, LZ, and HC took part in collecting samples and extracting data. XZ and ZH were responsible for the schematic diagram within this article and revised the manuscript. ZY and YL provided professional support and revised the manuscript. All authors read and approved the final manuscript.

## Funding

This work was supported by grants from Innovative Research Group Project of the National Natural Science Foundation of China (31921003) and Key Project of Chongqing Medical Scientific Research Project (Joint Project of Chongqing Health Commission and Science and Technology Bureau) (2022ZDXM002).

## Conflict of interest

The authors declare that the research was conducted in the absence of any commercial or financial relationships that could be construed as a potential conflict of interest.

## Publisher’s note

All claims expressed in this article are solely those of the authors and do not necessarily represent those of their affiliated organizations, or those of the publisher, the editors and the reviewers. Any product that may be evaluated in this article, or claim that may be made by its manufacturer, is not guaranteed or endorsed by the publisher.

## Abbreviations

AI: artificial intelligence
AUC: area under curve
CI: confidence interval
CT: computer tomography
DOC: disorders of consciousness
EEG: electroencephalogram
FOUR: full outline of unresponsiveness
HU: Hounsfield unit
ICH: intracerebral hemorrhage
MCS: minimally conscious state
MRI: magnetic resonance imaging
PBH: primary brainstem hemorrhage
P–P plots: probability-probability plots
ROC: receiver operating characteristics
SE: standard error

## References

[1] Chen P Yao H Tang X Wang Y Zhang Q Liu Y. Management of primary brainstem hemorrhage: A review of outcome prediction, surgical treatment, and animal model. Dis Markers. (2022) 2022:4293590. doi: 10.1155/2022/4293590, PMID: 35864996 PMC9296309

[2] Chen DY Tang YX Nie H Zhang P Wang WZ Dong Q. Primary brainstem hemorrhage: A review of prognostic factors and surgical management. Front Neurol. (2021) 12:727962. doi: 10.3389/fneur.2021.727962, PMID: 34566872 PMC8460873

[3] Kondziella D Bender A Diserens K van Erp W Estraneo A Formisano R. European academy of neurology guideline on the diagnosis of coma and other disorders of consciousness. Eur J Neurol. (2020) 27:741–56. doi: 10.1111/ene.14151, PMID: 32090418

[4] Zheng ZS Reggente N Lutkenhoff E Owen AM Monti MM. Disentangling disorders of consciousness: Insights from diffusion tensor imaging and machine learning. Hum Brain Mapp. (2017) 38:431–43. doi: 10.1002/hbm.23370, PMID: 27622575 PMC6867135

[5] Jang SH Kwon YH. The relationship between consciousness and the ascending reticular activating system in patients with traumatic brain injury. BMC Neurol. (2020) 20:375. doi: 10.1186/s12883-020-01942-7, PMID: 33054716 PMC7556972

[6] Chen Y Xu W Wang L Yin X Cao J Deng F. Transcranial Doppler combined with quantitative EEG brain function monitoring and outcome prediction in patients with severe acute intracerebral hemorrhage. Crit Care. (2018) 22:36. doi: 10.1186/s13054-018-1951-y, PMID: 29463290 PMC5820804

[7] Gomez LA Shen Q Doyle K Vrosgou A Velazquez A Megjhani M. Classification of level of consciousness in a neurological ICU using physiological data. Neurocrit Care. (2022) 38:118–28. doi: 10.1007/s12028-022-01586-0, PMID: 36109448 PMC9935697

[8] Goldfne AM Victor JD Conte MM Bardin JC Schif ND. Determination of awareness in patients with severe brain injury using EEG power spectral analysis. Clin Neurophysiol. (2011) 122:2157–68. doi: 10.1016/j.clinph.2011.03.022, PMID: 21514214 PMC3162107

[9] Cruse D Chennu S Chatelle C Bekinschtein TA Fernández-Espejo D Pickard JD. Bedside detection of awareness in the vegetative state: A cohort study. Lancet. (2011) 378:2088–94. doi: 10.1016/S0140-6736(11)61224-5, PMID: 22078855

[10] Stender J Gosseries O Bruno MA Charland-Verville V Vanhaudenhuyse A Demertzi A. Diagnostic precision of PET imaging and functional MRI in disorders of consciousness: A clinical validation study. Lancet. (2014) 384:514–22. doi: 10.1016/S0140-6736(14)60042-8, PMID: 24746174

[11] Fernández-Espejo D Norton L Owen AM. The clinical utility of fMRI for identifying covert awareness in the vegetative state: A comparison of sensitivity between 3T and 1.5T. PLoS One. (2014) 9:e95082. doi: 10.1371/journal.pone.0095082, PMID: 24733575 PMC3986373

[12] Sitt JD King J-R El Karoui I Rohaut B Faugeras F Gramfort A. Large scale screening of neural signatures of consciousness in patients in a vegetative or minimally conscious state. Brain. (2014) 137:2258–70. doi: 10.1093/brain/awu141, PMID: 24919971 PMC4610185

[13] Engemann DA Raimondo F King JR Rohaut B Louppe G Faugeras F. Robust EEG-based cross-site and cross-protocol classification of states of consciousness. Brain. (2018) 141:3179–92. doi: 10.1093/brain/awy251, PMID: 30285102

[14] Tan H Gu Y Yu H Hu P Zhang Y Mao W. Total-body PET/CT: Current applications and future perspectives. AJR Am J Roentgenol. (2020) 215:325–37. doi: 10.2214/AJR.19.22705, PMID: 32551910

[15] Han JW Zheng HF Cui Y Sun LD Ye DQ Hu Z. Genome-wide association study in a Chinese Han population identifies nine new susceptibility loci for systemic lupus erythematosus. Nat Genet. (2009) 41:1234–7. doi: 10.1038/ng.472, PMID: 19838193

[16] Chinese Society of Neurology. Chinese stroke society Chinese guidelines for diagnosis and treatment of acute ischemic stroke 2018 (in Chinese). Chin J Neurol. (2018) 51:666–82. doi: 10.3760/cma.j.issn.1006-7876.2018.09.004

[17] Lee H Zhao X Song HK Wehrli FW. Self-navigated three-dimensional ultrashort echo time technique for motion-corrected skull MRI. IEEE Trans Med Imaging. (2020) 39:2869–80. doi: 10.1109/TMI.2020.2978405, PMID: 32149683 PMC7484857

[18] Meng FH Wang JH Zhang HT Li W. Artificial intelligence-enabled medical analysis for intracranial cerebral hemorrhage detection and classification. J Healthc Eng. (2022) 2022:2017223. doi: 10.1155/2022/2017223, PMID: 35356628 PMC8959996

[19] Chang JB Wang RZ Feng M. The application of artificial intelligence in clinical diagnosis and treatment of intracranial hemorrhage. Chin J Contemp Neurol Neurosurg. (2019) 19:622–6. doi: 10.3969/j.issn.1672-6731.2019.09.004

[20] Hemphill JC III Greenberg SM Anderson CS Becker K Bendok BR Cushman M. Guidelines for the management of spontaneous intracerebral hemorrhage: A guideline for healthcare professionals from the American heart association/American stroke association. Stroke. (2015) 46:2032–60. doi: 10.1161/STR.0000000000000069, PMID: 26022637

[21] Lan Z Richard SA Li H Chen M Chao Y. Spontaneous hypertensive brainstem hemorrhage: Does surgery benefit the severe cases? Interdiscip Neurosurg Adv Tech Case Manag. (2019) 15:66–70. doi: 10.1016/j.inat.2018.10.015

[22] Giacino JT Fins JJ Laureys S Schiff ND. Disorders of consciousness after acquired brain injury: The state of the science. Nat Rev Neurol. (2014) 10:99–114. doi: 10.1038/nrneurol.2013.279 24468878

[23] Behrouz R. Prognostic factors in pontine haemorrhage: A systematic review. Eur Stroke J. (2018) 3:101–9. doi: 10.1177/2396987317752729, PMID: 31008342 PMC6460408

[24] Wijdicks EFM. The ascending reticular activating system. Neurocrit Care. (2019) 31:419–22. doi: 10.1007/s12028-019-00687-7 30796756

[25] Barras CD Tress BM Christensen S MacGregor L Collins M Desmond PM. Recombinant activated factor VII intracerebral hemorrhage trial investigators. Density and shape as CT predictors of intracerebral hemorrhage growth. Stroke. (2009) 40:1325–31. doi: 10.1161/STROKEAHA.108.536888, PMID: 19286590

[26] Bradley WG Jr. MR appearance of hemorrhage in the brain. Radiology. (1993) 189:15–26. doi: 10.1148/radiology.189.1.8372185 8372185

[27] Keselman B Gdovinová Z Jatuzis D Melo TPE Vilionskis A Cavallo R. Safety and outcomes of intravenous thrombolysis in posterior versus anterior circulation stroke: Results from the safe implementation of treatments in stroke registry and meta-analysis. Stroke. (2020) 51:876–82. doi: 10.1161/STROKEAHA.119.027071, PMID: 31914885

[28] Deng LN Wu BO. 《Chinese guidelines for diagnosis and treatment of cerebral hemorrhage 2019》update key points and interpretation. Cardio Cerebrovasc Dis Prev Treat. (2021) 21:13–17, 34. doi: 10.3969/j.issn.1009-816x.2021.01.002

[29] Wang JW Lin Y Xiong JH Yu SP Wei W Yang XY. Evaluation of spontaneous intracerebral hemorrhage by using CT image segmentation and volume assessment based on deep learning. Chin J Radiol. (2019) 53:941–5. doi: 10.3760/cma.j.issn.1005-1201.2019.11.003

[30] Zhang Y Wang J Schnakers C He MH Luo H Cheng LJ. Validation of the Chinese version of the coma recovery scale-revised (CRS-R). Brain Inj. (2019) 33:529–33. doi: 10.1080/02699052.2019.1566832, PMID: 30663434

[31] Oge DD Arsava EM Pektezel MY Gocmen R Topcuoglu MA. Intracerebral hemorrhage volume estimation: Is modification of the ABC/2 formula necessary according to the hematoma shape? Clin Neurol Neurosurg. (2021) 207:106779. doi: 10.1016/j.clineuro.2021.106779, PMID: 34214866

[32] Cerebrovascular Surgery Group of Chinese Society of Neurosurgery, Chinese Medical Doctor Association. Chinese neurosurgical expert consensus on the diagnosis and treatment of primary brainstem hemorrhage. Natl Med J China. (2022) 102:1068–75. doi: 10.3760/cma.j.cn112137-20211228-02913

[33] Ziai WC Thompson CB Mayo S Nichol M Freeman WD Dlugash R. Intracranial hypertension and cerebral perfusion pressure insults in adult hypertensive intraventricular hemorrhage: Occurrence and associations with outcome. Crit Care Med. (2019) 47:1125–34. doi: 10.1097/CCM.0000000000003848, PMID: 31162192 PMC7490004

[34] Liu HB Lf W Jf H Ss W. Research progress on the application of CT value in neurosurgery. Chin J Minim Invasive Neurosurg. (2021) 26:234–6. doi: 10.11850/j.issn.1009-122X.2021.05.013

[35] Yan LK Liu HJ Li JY Yang F Yan CM Cao HZ. CT quantitative assessment of intracerebral hemorrhage for dynamic evaluating the volume, density and shape evolution. J Clin Radiol. (2010) 29:1159–61. doi: 10.13437/j.cnki.jcr.2010.09.022

[36] Estraneo A Masotta O Bartolo M Pistoia F Perin C Marino S. Multi-center study on overall clinical complexity of patients with prolonged disorders of consciousness of different etiologies. Brain Inj. (2021) 35:1–7. doi: 10.1080/02699052.2020.1861652, PMID: 33331792

[37] Gregório T Pipa S Cavaleiro P Atanásio G Albuquerque I Chaves PC. Original intracerebral hemorrhage score for the prediction of short-term mortality in cerebral hemorrhage: Systematic review and meta-analysis. Crit Care Med. (2019) 47:857–64. doi: 10.1097/CCM.0000000000003744, PMID: 30889025

[38] You C Tao CY. History, present and future of diagnosis and treatment of primary brainstem hemorrhage. Chin J Contemp Neurol Neurosurg. (2021) 21:71–5. doi: 10.3969/j.issn.1672-6731.2021.02.002

[39] Zheng RZ Qi ZX Wang Z Xu ZY Wu XH Mao Y. Clinical decision on disorders of consciousness after acquired brain injury: Stepping forward. Neurosci Bull. (2023) 39:138–62. doi: 10.1007/s12264-022-00909-7, PMID: 35804219 PMC9849546

[40] Tao C Li H Wang J You C. Predictors of surgical results in patients with primary pontine hemorrhage. Turk Neurosurg. (2016) 26:77–83. doi: 10.5137/1019-5149.JTN.12634-14.1, PMID: 26768872

[41] Shrestha BK Ma L Lan Z Li H You C. Surgical management of spontaneous hypertensive brainstem hemorrhage. Interdiscip Neurosurg. (2015) 2:145–8. doi: 10.1016/j.inat.2015.06.005

[42] Huang K Ji Z Sun L Gao X Lin S Liu T. Development and validation of a grading scale for primary pontine hemorrhage. Stroke. (2017) 48:63–9. doi: 10.1161/STROKEAHA.116.015326, PMID: 27932606

